# Odontogenic keratocysts in Nevoid basal cell carcinoma syndrome: a case report

**DOI:** 10.1186/1757-1626-2-9399

**Published:** 2009-12-24

**Authors:** Nooshin Mohtasham, Somayyeh Nemati, Shokoofeh Jamshidi, Ataollah Habibi, Masume Johari

**Affiliations:** 1 Department of Oral and Maxillofacial Pathology, Faculty of Dentistry and Dental Research Center, Mashhad University of Medical Sciences, Mashhad, Iran; 2 Department of Oral and Maxillofacial Radiology, Faculty of Dentistry and Dental Research Center, Mashhad University of Medical Sciences, Mashhad, Iran; 3 Department of Oral and Maxillofacial Pathology, Faculty of Dentistry and Dental Research Center, Hamedan University of Medical Sciences, Hamedan, Iran; 4 Department of Oral and Maxillofacial surgery, Faculty of Dentistry and Dental Research Center, Mashhad University of Medical Sciences, Mashhad, Iran; 5 Department of Oral and Maxillofacial Radiology, Faculty of Dentistry and Dental Research Center, Tabriz University of Medical Sciences, Tabriz, Iran

## Abstract

Nevoid basal cell carcinoma syndrome, a rare autosomal dominant disorder, comprises a number of abnormalities such as multiple nevoid basal cell carcinomas, skeletal abnormalities and multiple odontogenic keratocysts. Considering the rarity of this syndrome, we present a 12-year-old boy affected by this syndrome. He had multiple okcs, calcification of falx cerebri, bifid ribs, frontal bossing and hypertelorism. Characteristic cutaneous manifestation (nevoid basal cell carcinoma) was not present in this patient. The jaw cysts were treated with marsupialization then enucleation. The dental clinician may be the first to encounter and identify this syndrome, when the multiple cystlike radiolucencies are discovered on panoramic view.

## Background

Nevoid basal cell carcinoma syndrome (NBCCS), is an autosomal dominant disorder with a high degree of penetrance and a variable expressivity characterized by several developmental defects and predisposition to cancer. Prevalence of NBCCS ranges from 1:6000 subjects in England, and to 1:164000 in Australia with males and females equally affected [1,2].

The syndrome, first delineated by Gorlin and Goltz, is characterized by basal cell carcinoma, odontogenic keratocysts, palmar and/or plant pits and ectopic calcification of the falx cerebri [2-4]. These traits are considered major clinical diagnostic criteria. Basal cell carcinoma is reported in approximately 76% of NBCCS cases, affect primarily the face and back followed by chest[2]. Seventy-five percent of patients affected by NBCCS often show multiple and bilateral odontogenic keratocysts. They are mainly located in the premolar area, may displace teeth with consequent malocclusion and can be unilocular or multilocular with a preference for the mandible. Jaw cysts are often asymptomatic but occasionally they may present pain, swelling, intraoral drainage, visual disturbances or parasthesia, furthermore, they may cause pathologic fractures of the mandible or facial disturbances [2]. The dental clinician may be the first to encounter and identify this syndrome when multiple cyst like radiolucencies are discovered on radiographs of the jaws [4-7].

More than 100 minor criteria have been described for NBCCS but cardiac or ovarian fibroma, bifid ribs, macrocephaly, kyphoscoliosis, cleft palate and meduloblastoma are the most frequent [2]. Also, vertebral fusion, polydoctyly, frontal and temporoparietal bossing, a mild ocular hypetelorism and a mild prognathism can be showed in this syndrome. The Ellsworth-Howord test is used to differentiated NBCCS from other disease status[4].

Diagnosis of NBCCS may be difficult because of the variability of expressivity and because of different ages of onset for different traits of this disorder. Average age for diagnosis of NBCCS is 13 years while average age for detection of basal cell carcinoma is 20 years. The clinical expression of the syndrome varies among individuals within the same family and even more among families [2].

NBCCS seems to be caused by castitutional aberrations of gene PTCH mapped to the long arm of chromosome 9 locus 9q 22, 3-q31 with no heterogeneity. About58 to 60% of subjects fulfilling diagnostic criteria show the gene defect [2,8]. This syndrome starts to appear early in life usually after 5 years of age and before 30 years of age. The jaw cysts usually appearing earlier in life than solitary odontogenic keratocysts, also earlier than other manifestation of this syndrome and recurrence of them is greater than solitary odontogenic keratocysts[3]. Histopathologically, the cysts of this syndrome is always odontogenic keratocysts[9]. Considering the rarity of this syndrome we present a young subject affected by NBCCS.

## Case presentation

A 12-year-old boy was referred to the Dental School of Mashhad University of Iran, requiring treatment for two large osteolytic lesions, with a complaint of bilateral swelling of the maxilla. About one month prior to this referral he had swelling of the maxilla. A full body clinical examination revealed bilateral swelling of the buccal aspect of the posterior region of the maxilla, in which the lesions were soft and rubbery in palpation. Also frontal bossing and hypertelorism were evident. The panoramic radiography depicted two large radiolucent lesions. With these findings, we suspected multiple jaw cysts associated with NBCCS. Thus the patient underwent a posterior anterior(PA) skull view and chest radiography.

**Figure 1:**
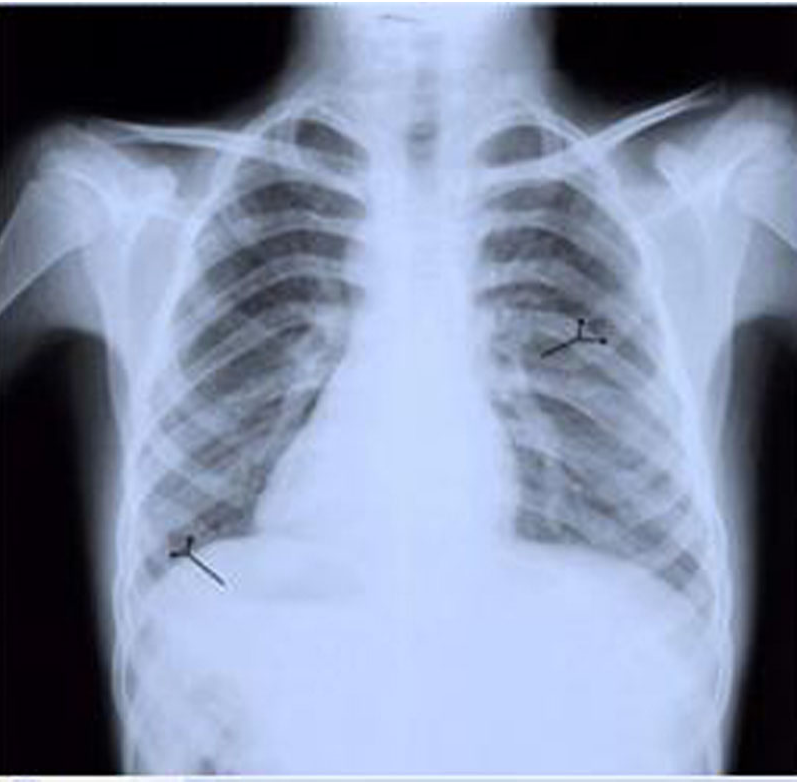
**Chest radiography: Note the bifid ribs in both sides**.

The chest radiography depicted two bifid ribs in both sides(Figure 1).

**Figure 2:**
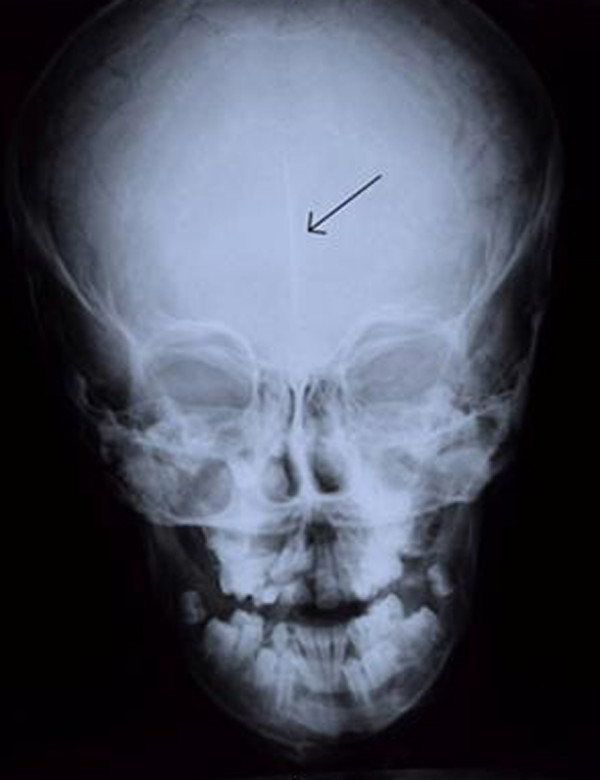
**Posterior-Anterior skull view: note the linear faint calcification of falx cerebri**.

The PA skull view, revealed linear faint calcification (Figure 2).

**Figure 3:**
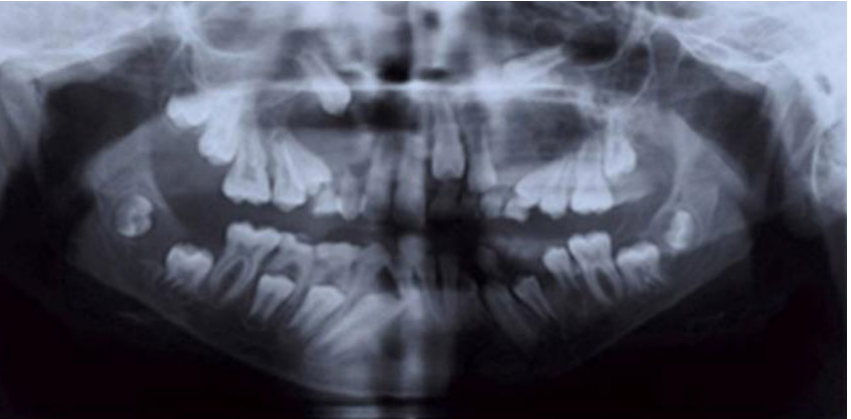
**Panoramic radiography: shows three odontogenic keratocyst (two lesions in canine regions of maxilla and one pericoronal lesion associated with right third molar of mandible**.

Panoramic radiography showed two well defined unilocular radiolucencies in maxillary canine regions. One lesion extended from the distal aspect of the maxillary left lateral incisor to the distal of the left first molar and another from the mesial aspect of the maxillary left central incisor to the distal of the right first premolar. Both lesions displaced canine teeth superiorly. Also a pericoronal radiolucency associated with the mandibular right third molar was observed (Figure 3). The presence of minor and major criteria allowed the diagnosis of NBCCS. The patient was referred to the Center of Oral and Maxillofacial Surgery. He then underwent incisional biopsy of jaw lesions.

**Figure 4:**
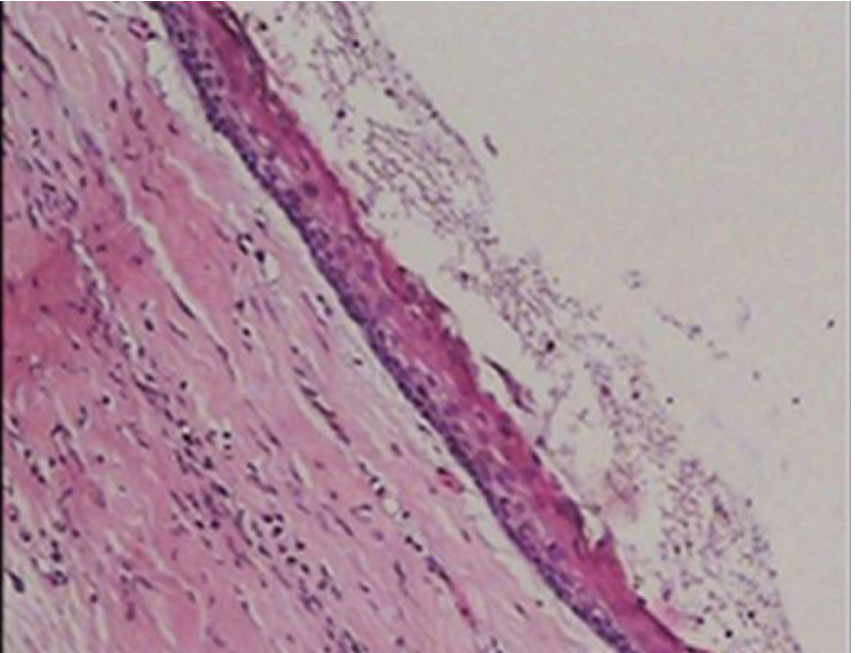
**Histopathologic view: The epithelial lining of the odontogenic keratocyst with a hyperchromatic and palisaded basal cell layer and corrugated parakeratotic surface (100× magnification-Hematoxylin & Eosin staining)**.

**Figure 5:**
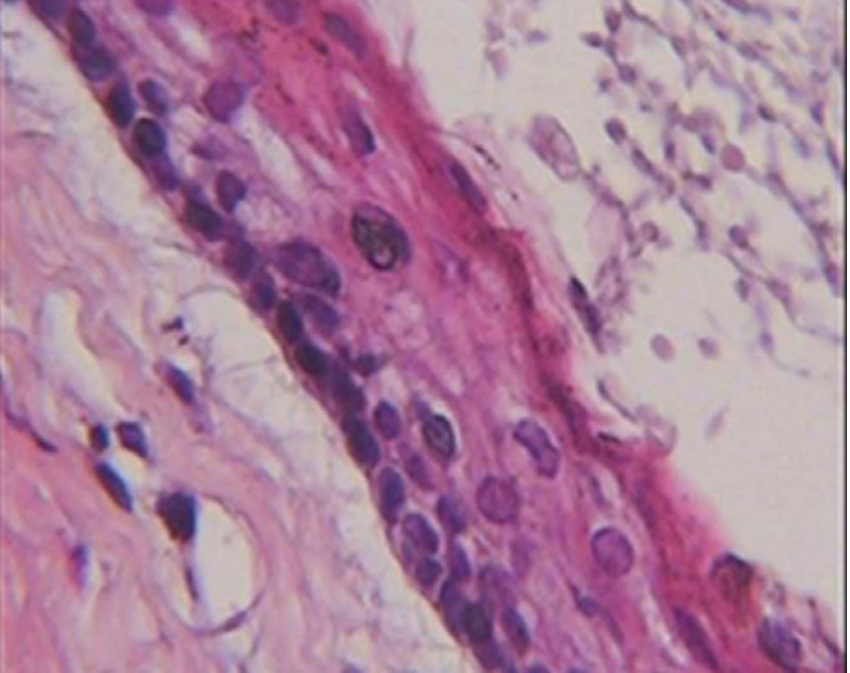
**Histopathologic view: The epithelial lining of the odontogenic keratocyst with a hyperchromatic and palisaded basal cell layer and corrugated parakeratotic surface (400× magnification-Hematoxylin & Eosin staining)**.

The histological analysis showed that the epithelial lining of cyst composed of a uniform layer of stratified squamous epithelium, the luminal surface show flattened parakeratotic epithelial cell, which exhibited a corrugated appearance. The basal layer was composed of a palisaded layer of cuboidal or columnar epithelial cells. The interface of epithelium and connective tissue was flat and the thin fibroma wall was devoided of any inflammatory infiltration. This confirmed the diagnosis of odontogenic keratocysts (Figures 4 and 5). Consequently, three lesions were treated with marsupialization then enucleation. Because of a high recurrence rate of odontogenic keratocysts in this syndrome than solitary odontogenic keratocysts, the aggressive treatment is necessary. It is reasonable to examine the patient yearly for new and recurrent cysts. A panoramic film serves as an adequate screening film. In present case, one year after surgery, there was no evidence of recurrence of jaw cysts.

## Conclusions

NBCCS is a hereditary complex of the abnormalities transmitted as an autosomal dominant trait. Characteristic features of NBCCS embrace varying manifestations of cutaneous and skeletal abnormalities and frequently ectopic calcification. The most notable and characteristic cutaneous manifestation is nevoid basal cell carcinoma. Skeletal abnormalities may include multiple cysts of the jaws that generally develop at an earlier age than basal cell carcinoma, bifid ribs, synostosis of the ribs, kyphoscolliosis, vertebral fusion, polyadactyly, frontal and temporoparietal bossing, a mild ocular hypertelorism and a mild prognathism [5,9]. Considering the rarity of this syndrome, a young subject affected by NBCCS was presented in this article.

Some criteria that included falx cerebri calcification, frontal bossing, hypertelorism, multiple odontogenic keratocysts, bifid ribs were compatible with the diagnostic criteria of NBCCS and it allowed a diagnosis of this syndrome. Characteristic cutaneous manifestation (nevoid basal cell carcinoma) was not present in this patient because the jaw cysts usually appearing earlier in life than basal cell carcinomas.

In conclusion, it is important to note that the dental clinician may be the first to encounter and identify this syndrome when the multiple cystlike radiolucencies are discovered on radiographs of the jaws.

## Abbreviations

OKCs: Odontogenic Keratocysts; PA: Posterior Anterior.

## Consent

Written informed consent was obtained from the patient for publication of this case report and accompanying images. A copy of the written consent is available for review by the Editor-in-Chief of this journal

## Competing interests

The authors declare that they have no competing interests.

## Authors' contributions

NM: Histopathology diagnosis, writing the manuscript.

MJ: Performance and interpretation of radiographs, writing the manuscript, correspondent author.

SJ: Histopathology diagnosis, writing the manuscript.

SN: Performance and interpretation of radiographs, writing the manuscript.

AH: Performance of surgery and writing the manuscript.

Also, all of authors read and approved the final manuscript.
